# Identification of *Streptococcus pneumoniae* Serotype 11E, Serovariant 11Av and Mixed Populations by High-Resolution Magic Angle Spinning Nuclear Magnetic Resonance (HR-MAS NMR) Spectroscopy and Flow Cytometric Serotyping Assay (FCSA)

**DOI:** 10.1371/journal.pone.0100722

**Published:** 2014-06-26

**Authors:** Romina Camilli, Brady L. Spencer, Monica Moschioni, Vittoria Pinto, Francesco Berti, Moon H. Nahm, Annalisa Pantosti

**Affiliations:** 1 Department of Infectious, Parasitic and Immune-mediated Diseases, Istituto Superiore di Sanità, Rome, Italy; 2 Department of Pathology, University of Alabama at Birmingham, Birmingham, Alabama, United States of America; 3 Novartis Vaccines & Diagnostics, Siena, Italy; 4 Department of Microbiology, University of Alabama at Birmingham, Birmingham, Alabama, United States of America; Centers for Disease Control & Prevention, United States of America

## Abstract

**Background:**

Recent studies have identified *Streptococcus pneumoniae* serotype 11E and serovariant 11Av among isolates previously typed as 11A by classical serotyping methods. Serotype 11E and serovariant 11Av differ from serotype 11A by having totally or partially inactive *wcjE*, a gene in *cps* locus coding for an O-acetyl transferase. Serotype 11E is rare among carriage isolates but common among invasive isolates suggesting that it survives better during invasion. Aim of this work was to investigate the epidemiology of serotype 11A in a pneumococcal collection using a new serotyping approach based on High-Resolution Magic Angle Spinning Nuclear Magnetic Resonance (HR-MAS NMR) spectroscopy to distinguish serotypes 11A and 11E.

**Methods:**

A collection of 48 (34 invasive and 14 carriage) *S. pneumoniae* isolates from Italy, previously identified as serotype 11A by the Quellung reaction, were investigated by *wcjE* sequencing, HR-MAS NMR spectroscopy and the reference flow cytometric serotyping assay (FCSA) based on monoclonal antibodies.

**Results:**

HR-MAS NMR spectra from serotypes 11A and 11E showed different NMR peaks indicating that HR-MAS NMR could be used to distinguish these serotypes, although HR-MAS NMR could not distinguish serotype 11Av from serotype 11E unambiguously. Thirty-eight isolates were confirmed to be serotype 11A, 8 isolates with a mutated *wcjE* were serotype 11E, 1 isolate belonged to serovariant 11Av, and 1 isolate was a mixed population 11A/11Av. All 11E isolates were identified among invasive isolates.

**Conclusions:**

We proved that HR-MAS NMR can be of potential use for pneumococcal serotyping. The detection of serotype 11E among invasive isolates in our collection, supports previous epidemiological studies suggesting that mutations in *wcjE* can represent a mechanism promoting pneumococcal survival during invasion. The discovery of a spectrum of immunochemical diversity within established serotypes should stimulate efforts to develop new serotyping approaches.

## Introduction

*Streptococcus pneumoniae* is an important human pathogen responsible for severe life-threatening infections, including meningitis and pneumonia, especially in children and elderly people. The capsular polysaccharide (CPS), which provides a barrier against host cell-mediated phagocytosis, represents the major virulence factor of *S. pneumoniae* [1]. At least 90 different pneumococcal capsular types or serotypes have been recognized on the basis of antigenic, biochemical, and genetic characteristics [2], [3]. Serotypes can be differentiated by the capsular swelling or Quellung reaction, using specific rabbit antisera [4] and partial cross-reactions can be used to cluster serotypes into serogroups, with each serogroup consisting of 2–5 serotypes. Serogroup 11 had been known to contain 5 serotypes (11F, 11A, 11B, 11C, 11D), sharing substantial homologies in the structure of the CPS, that consists of a linear tetrasaccharide backbone with different degrees of O-acetylation, and in the capsular polysaccharide synthesis locus (*cps*), differing mainly in O-acetyltransferase genes (*wcwR*, *wcwC*, *wcjE*) [5]. Serotype 11A is one of the most common serotypes both in human carriage and in infection [6], [7] and is associated with two distinct *cps* alleles designated 11A-1 and 11A-2 [8]. 11A-1 corresponds to the *cp*s allele recovered in the majority of the 11A strains sequenced so far [9]–[11], and is identical to the 11D *cps* locus described by Bentley *et al.* [2], with the exception of a single nucleotide polymorphism (SNP) in *wcrL*, a gene coding for a putative glycosyltransferase [10], [12]; 11A-2 corresponds to the *cps* allele reported by Bentley *et al.* for serotype 11A [2] but is only found in a minority of serotype 11A isolates [8].

The improvements of serotyping techniques such as those employing monoclonal antibodies [13] allowed the identification of new serotypes within known serogroups, such as 6, 11 and 20 [9], [14], [15]. Within serogroup 11, a new serotype designated 11E, was identified among isolates previously serotyped as 11A by the Quellung reaction [9]. Serotype 11E is characterized by an 11A *cps* with a mutated or disrupted *wcjE*, a gene coding for an O-acetyl transferase [9]. In some cases, the mutated WcjE was partially functional and the strains with partially functional WcjE have been labeled 11A serovariants (11Av) [8]. These mutations lead to a structural modification of the CPS, which differs from that of 11A for the total or partial absence of an O-acetyl group at C_6_ position β-Gal [8], [16]. The majority of serotype 11E isolates identified so far contains distinct and unrelated *wcjE* mutations, suggesting that the isolates arose independently from serotype 11A progenitors [9]. An epidemiological study showed that serotype 11E is rare among nasopharyngeal carriage isolates but is common among invasive isolates [17]. This observation suggests that serotype 11A strains that have mutated into 11E survive better during invasion into deeper tissues. A recent study has demonstrated that a host immune factor, namely ficolin-2, recognizes serotype 11A but not serotype 11E, possibly selecting for micro-evolution of 11A into 11E or 11Av [18].

Because of the novelty of the situation, confirmation of the above findings is important. Yet, serotype 11E and serovariant 11Av are currently distinguishable only by the Flow Cytometric Serotyping Assay (FCSA) based on monoclonal antibodies [8]. Recently, we used High-Resolution Magic Angle Spinning Nuclear Magnetic Resonance (HR-MAS NMR) spectroscopy to demonstrate lack of CPS in two *S. pneumoniae* isolates with point mutations in the *cps* gene *wchA* [19]. HR-MAS NMR differs from high-resolution liquid phase NMR (HR-NMR), which is the golden standard method for accurate CPS structural characterization and is the validated procedure for pneumococcal polysaccharide vaccine release [20], being a simpler and faster procedure that does not require polysaccharide purification. Aim of this study was to harness HR-MAS NMR as a new way to distinguish 11E from 11A, in order to study the epidemiology of serotype 11A, 11E and 11Av in a set of pneumococcal isolates from Italy.

## Materials and Methods

### Pneumococcal isolates

All the 11A isolates (n = 48) available in the pneumococcal collection of the Istituto Superiore di Sanità were included in the study. Thirty-four invasive isolates were obtained in the framework of the Nationwide Surveillance of Invasive Bacterial Diseases (http://www.simi.iss.it) in the years from 1997 to 2011; 13 isolates were obtained from a carriage study in children conducted in 2011–2012 [21], and 1 isolate was obtained from a tracheal aspirate from a cystic fibrosis patient in 2012. The isolates were assigned to serotype 11A by Latex agglutination and the Quellung reaction using antisera produced by the Statens Serum Institut (Copenhagen, Denmark).

Serotype 11A strain SSISP11A/2 provided by the Statens Serum Institut, and serotype 11E strain MNZ269 [9], were used as controls. Cultures were performed on Columbia agar plates supplemented with 5% sheep blood at 37°C in 5% CO_2_. For genomic DNA extraction, isolates were grown in Todd Hewitt Broth (THB) (Oxoid Microbiology Products, Basingstoke, UK). For NMR spectroscopy analysis, cultures were grown in THB supplemented with 0.5% (w/w) yeast extract (THYE) to OD_600_ 0.25.

### Polymerase Chain Reaction (PCR) and sequencing of the *wcjE* gene

Genomic DNA from pneumococcal isolates was extracted using QIAmp DNA mini kit (Qiagen, Hilden, Germany), following the manufacturer's instructions. To amplify the *wcjE* gene, a PCR reaction was performed using the primer pairs wcjE_2, (5′-TGATTGTCCTCTCCAGATG-3′), and wcjE_3, (5′-GGTAAGAATGTATTAGATTTAGC-3′); to amplify *wcjE* and its upstream region the primer pair 5309 and 5310 were used [15]. The amplified products were purified using QIAquick PCR Purification kit (Qiagen, Hilden, Germany) and sequenced. DNA sequences were analyzed by Lasergene version 5 software (DNASTAR, Madison, WI, USA).

### Flow cytometric serotyping assay (FCSA)

Serotypes 11A and 11E were distinguished by binding to two monoclonal antibodies, Hyp11AM1 (IgM) and Hyp11AG2 (IgG) by FCSA, as described previously [8]. Briefly, bacteria (1×10^7^ CFU/mL) were incubated with 1∶10 and 1∶100 dilutions of Hyp11AM1 and Hyp11AG2 hybridoma supernatants, respectively. Bound antibodies were detected with RPE-conjugated α-mouse IgG and PE-Cy7-conjugated α-mouse IgM. Stained bacteria were studied with a FACSCalibur (BD Biosciences, San Jose, CA, USA) and data was analyzed with FCS Express (De Novo Software, Los Angeles, CA, USA).

### CPS detection by high-resolution liquid phase NMR (HR-NMR) and high-resolution magic angle spinning NMR (HR-MAS NMR) spectroscopy

To investigate the HR-NMR profile of the purified 11A polysaccharide, approximately 1 mg of 11A polysaccharide (Merck, Darmstadt, Germany) was dissolved in 0.75 ml deuterium oxide (D_2_O; Sigma-Aldrich, St. Louis, MO, USA) and placed in a 5 mm NMR tube (Wilmad Lab-Glass, Vineland, NJ, USA), and its ^1^H NMR spectrum was recorded as previously described [19]. To investigate the HR-NMR profiles of de-O-Acetylated polysaccharides, the cell pellets of strains AP200 (11A), AP366 (11A), and AP446 (11E), were re-suspended in 0.75 mL D_2_O (Sigma-Aldrich) and inserted into a 5 mm NMR tubes (Wilmad). ^1^H NMR spectra were collected on the soluble fraction of polysaccharides naturally released from the cells before and after the addition of 30 µL of 5 M NaOD to get a final concentration of 200 mM.

For HR-MAS NMR, bacterial cells grown in THYE were inactivated by 1% (v/v) formaldehyde treatment and washed three times with PBS in D_2_O. Approximately 50 µl of compact cell pellet was placed into a 50 µl Kel-F disposable insert and then in a 4 mm MAS ZrO_2_ rotor (Bruker). Data from all NMR experiments, either performed in liquid phase (HR-NMR) or in heterogeneous phase (HR-MAS NMR), were recorded as previously described [19].

## Results

### *wcjE* genotypes

Analysis of *wcjE* sequences revealed that 36 out of 48 pneumococcal isolates carried an intact *wcjE*, which could be readily classified into 11A-1 or 11A-2 allele as previously described [15]. Among the 36 isolates, 32 carried a *wcjE* 11A-1 allele and 3 carried a *wcjE* 11A-2 allele. In one isolate (PER155), *wcjE* was 99% identical to the 11A-1 allele due to a silent SNP (Table 1). Eleven out of 48 isolates had a mutated *wcjE* and all were invasive isolates. Three isolates had a distinct SNP in *wcjE*, leading to an amino acid change that should not disrupt WcjE protein expression. The other 8 isolates had a disrupted *wcjE*: 5 isolates had *wcjE* disrupted by a transposable element (IS*1515* or IS*1167*) and 3 isolates showed unique mutations in *wcjE* (duplications in two isolates and nonsense SNP in one) resulting in a premature stop codon or in a later start codon (Table 1).

**Table 1:** *wcjE* sequencing results and serotype assignment of the 11A pneumococcal isolates by FCSA and HR-MAS NMR.

Isolates	Year	Source	*wcjE* allele	*wcjE* mutations	Serotype	
					FCSA	NMR^a^
PN074	1998	CSF	11A-1	-	11A	11A
SP135	1999	CSF	11A-1	-	11A	nd
SP174	1999	Blood	11A-1	-	11A	nd
PNS31	2000	Blood	11A-1	-	11A	nd
PN174	2001	CSF	11A-1	-	11A	11A
PN175	2001	CSF	11A-1	-	11A	nd
AP156	2002	CSF	11A-1	-	11A	nd
AP053	2002	Blood	11A-1	-	11A	nd
AP200	2003	CSF	11A-1	-	11A	11A
PN223	2004	CSF	11A-1	-	11A	nd
AP324	2006	Blood	11A-1	-	11A	nd
PN301	2008	CSF	11A-1	-	11A	nd
AP649	2008	Blood	11A-1	-	11A	nd
PER191	2008	Blood	11A-1	-	11A	11A
AP613	2008	Blood	11A-1	-	11A	11A
PN436	2009	Blood	11A-1	-	11A	11A
AP713	2009	Blood	11A-1	-	11A	11A
PN609	2011	Blood	11A-1	-	11A	nd
BO034	2012	Nasopharynx	11A-1	-	11A	nd
BO035	2012	Nasopharynx	11A-1	-	11A	nd
BO045	2012	Nasopharynx	11A-1	-	11A	nd
BO062	2012	Nasopharynx	11A-1	-	11A	nd
BO175	2012	Nasopharynx	11A-1	-	11A	nd
BO204	2012	Nasopharynx	11A-1	-	11A	nd
MI027	2012	Nasopharynx	11A-1	-	11A	nd
MI030	2012	Nasopharynx	11A-1	-	11A	nd
MI122	2012	Nasopharynx	11A-1	-	11A	nd
MI243	2012	Nasopharynx	11A-1	-	11A	nd
MI244	2012	Nasopharynx	11A-1	-	11A	nd
MI294	2012	Nasopharynx	11A-1	-	11A	nd
PFCO35	2012	Tracheal aspirate	11A-1	-	11A	11A
AP366	2006	Blood	11A-2	-	11A	11A
PN695	2011	Blood	11A-2	-	11A	nd
PN694	2011	Blood	11A-2	-	11A	11A
MI132	2012	Nasopharynx	11A-1	-	11Av/11A	11A
PER155	2008	Blood	11A-1	1 silent SNP: C147T	11A	11A
PER098	2007	Blood	11A-1	1 missense SNP: C278T, S93L	11A	11A
AP508	2007	Pleural effusion	11A-2	1 missense SNP: G189A, G62R	11A	11A
AP789	2010	Blood	11A-1	1 missense SNP: C844T, H282Y	11E	11E
SP304	2000	Blood	11A-1	Insertion of IS*1515* resulting in the direct repeat of sequence 965-TATT-968	11E	11E
AP278	2005	Blood	11A-1	Insertion of IS*1515* resulting in the direct repeat of sequence 965-TATT-968	11E	11E
AP344	2006	Blood	11A-1	Insertion of IS*1515* resulting in the direct repeat of sequence 965-TATT-968	11E	11E
PN259	2007	CSF	11A-1	Insertion of IS*1515* resulting in the direct repeat of sequence 466-GTTAGT-471	11E	11E
AP446	2006	Blood	11A-2	Insertion of IS*1167* resulting in the direct repeat of sequence 269-TTTTTTAT-276	11E	11E
PN066	1997	CSF	11A-1	Duplication of bp 181-TGTTTTTTGCGTTA-194 resulting in a premature stop codon (226-TGA-228)	11E	11E
SP335	2000	Blood	11A-1	Duplication of bp 427-TGGTTTA-433 resulting in a premature stop codon (457-TAA-459)	11E	11E
AP/PT136	2003	Blood	11A-1	G24A mutation resulting in a premature stop codon (22-TGA-24) and in a later start codon (643-ATG-645)	11Av	11E
PN182	2001	CSF	11A-1	100% *id* 11A-1/Duplication of 325-GA-326 resulting in a premature stop codon (352-TAA-354)	11A	11A

CSF: cerebrospinal fluid.

nd: not determined.

a HR-MAS NMR.

In one (PN182) of the 48 isolates, sequence analysis showed partially superimposed sequences. Sequencing of 10 distinct colonies from PN182 frozen stock revealed that 2 colonies (20%) showed a 2-nt duplication (325-GA-326) in *wcjE*, leading to a premature stop codon. The duplication was shown to be a stable character when colonies were cultured for at least 6 passages.

### Determination of capsule phenotypes by FCSA

FCSA analysis confirmed that all the isolates with an intact *wcjE* belong to serotype 11A (Table 1). The 8 isolates with a disrupted *wcjE* (all invasive isolates) were identified as belonging to serotype 11E, except isolate AP/PT136 that was identified as 11Av. One invasive isolate with an amino acid change in WcjE (AP789) was assigned to serotype 11E. Overall, 8 out of 34 invasive isolates (23.5%) belonged to serotype 11E. Serotype 11E was not found among carriage isolates. A mixed population 11Av/11A was identified in one carriage isolate (MI132) (Table 1).

### Determination of capsule phenotypes by HR-MAS NMR

**Figure 1:**
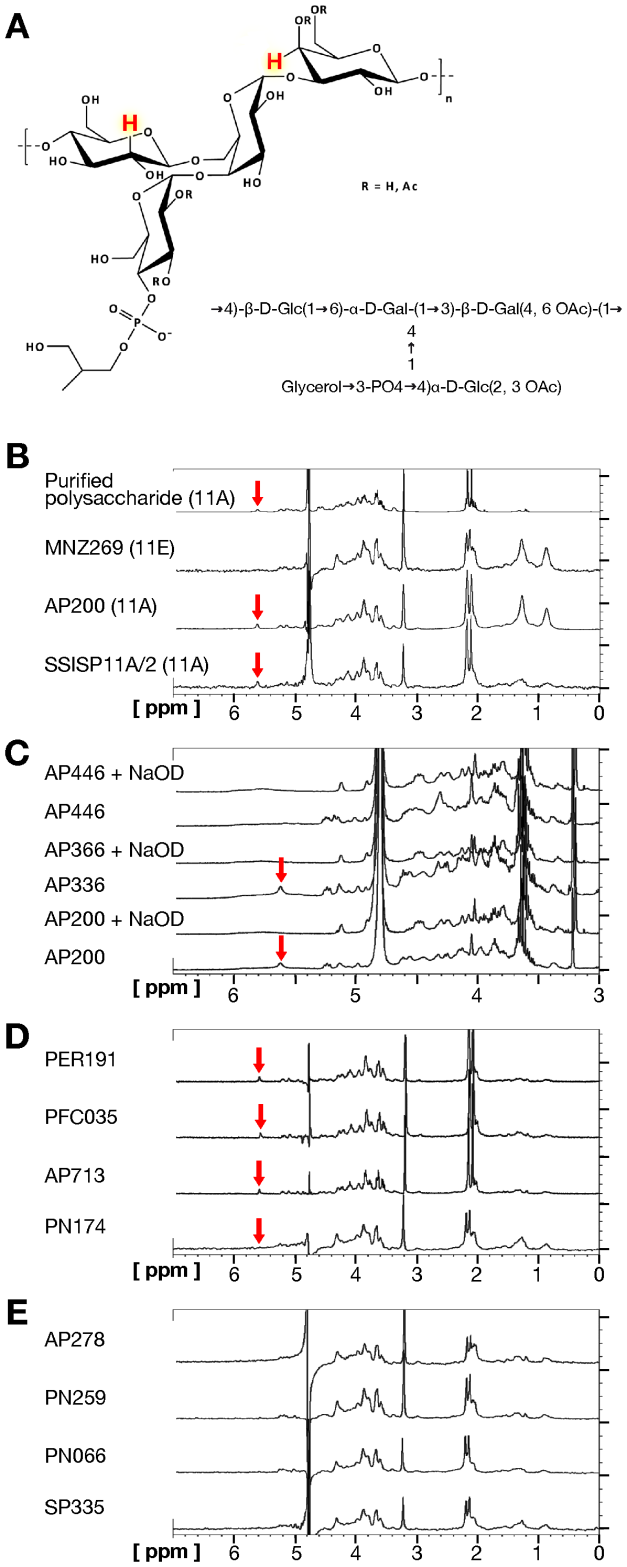
HR-NMR and HR-MAS NMR analyses. Panel A: Backbone of *S. pneumoniae* serotype 11A and 11E polysaccharide. The proton at position C_4_ of β-Gal residue O-acetylated at both position C_4_ and C_6_ (H_4_^βGal-4,6OAc^), which was used as probe for 11A strains detection, and the proton at position C_2_ of the β-Glc residue (H_2_^βGlc^), which was used for peak normalization, are labeled. Panel B: Proton NMR spectrum in liquid phase of *S. pneumoniae* purified 11A polysaccharide and proton HR-MAS NMR spectra in heterogeneous phase of *S. pneumoniae* 11A (SSISP 11A/2, AP200) and 11E (MNZ269) isolates. Red arrows indicate the H_4_^βGal-4,6OAc^ signal at 5.6 ppm. Panel C. Proton NMR spectra in liquid phase of *S. pneumoniae* 11A (AP366, AP200) and 11E (AP446) supernatants before and after treatment with NaOD. Red arrows indicate the H_4_^βGal-4,6OAc^ signal at 5.6 ppm. Panel D: Proton HR-MAS NMR spectra in the heterogeneous phase of *S. pneumoniae* 11A isolates (PER191, PFC035, AP713, PN174). Red arrows indicate the H_4_^βGal-4,6OAc^ signal at 5.6 ppm. Panel E: Proton HR-MAS NMR spectra in the heterogeneous phase of *S. pneumoniae* 11E isolates (AP278, PN259, PN066, SP335).

Analysis of ^1^H NMR spectra obtained from purified polysaccharide 11A, and from reference 11A (SSISP11A/2) and 11E (MNZ269) isolates revealed specific peaks discriminating 11A from 11E. The HR-MAS NMR spectroscopy method used in this study identified a peak at 5.6 ppm, assigned to the proton at position C_4_ of the β-Gal residue as the 11A probe signal (Figure 1, panel A and panel B). This peak appeared low-field shifted due to the presence of O-acetyl groups at both position C_4_ and C_6_ of the β-Gal residue (H_4_^βGal-4,6OAc^) in 11A while it was not detectable in 11E (Figure 1, panel A). The proton signal at position C_2_ of the β-Glc residue (H_2_^βGlc^) at 3.4 ppm was used for peak normalization (Figure 1, panel A).

^1^H spectra collected on the native and de-O-acetylated 11A and 11E polysaccharides demonstrated that the discriminating H_4_^βGal-4,6OAc^ peak at 5.6 ppm disappeared after basic treatment with NaOD (Figure 1, Panel C), as reported by Calix *et al.* [8]. The complete cleavage of O-acetyl groups at position C_2_ and C_3_ of α-Glc, and at position C_4_ and C_6_ of β-Gal reduced the peaks complexity of ^1^H NMR profiles of both 11A and 11E isolates which, consequently, appeared superimposable.

Proton HR-MAS NMR spectra directly recorded on inactivated cells in heterogeneous phase were blindly collected for a subset of isolates (n = 24), chosen on the basis of FCSA results and including all 11E and 11Av isolates, and 13 serotype 11A isolates (Table 1). NMR results were in line with FCSA results, being able to distinguish serotypes 11A and 11E isolates (Figure 1, panels D and E). However, NMR was not efficient in discriminating the intermediate serovariant 11Av since isolate AP/PT136 was identified as 11E, neither in detecting different subpopulations in the same strain, since MI132 (11A/11Av) was identified as serotype 11A (Table 1).

## Discussion

The discovery of new serotypes [9], [14], [15], of different serovariants [8], and of hybrid serotypes [22], highlighted that capsule expression in *S. pneumoniae* is more complex than previously recognized. *S. pneumoniae* appears to be a very adaptable bacterium, able to modify its surface polysaccharides in response to the host's immune selective pressure. This mechanism has likely contributed to the evolution of a broad variety of immunochemically distinct pneumococcal capsular types.

The new serotype 11E was discovered among isolates previously recognized as belonging to serotype 11A, carrying a *cps* with a disrupted *wcjE* [9]. According to these studies approximately 25% of serotype 11A isolates from invasive diseases should be re-classified as serotype 11E [15]. Recent data demonstrated that serotypes 11A and 11E actually represent the two extremes of an antigenic spectrum that also includes the intermediate variant 11Av [8], which expresses variable degree of O-acetylation on β-Gal C_6_ due to variable reduction in WcjE activity. Hence, the genetic analysis of *wcjE* is not sufficient to discriminate this spectrum and only FCSA, a serotyping method based on flow-cytometry and monoclonal antibodies, can detect a full (11A), null (11E) or partial (11Av) expression of the O-acetyl 11A-specific epitope.

In this study we tested the feasibility of using HR-MAS NMR to discriminate serotype 11A and 11E, since this method had been previously successfully used in a study on spontaneous pneumococcal capsular mutants [19]. HR-MAS NMR uses a rapidly spinning sample tube and allows detection of different CPS on inactivated bacterial cells without purification of the polysaccharide, which is necessary for liquid phase HR-NMR. Yet, HR-MAS NMR requires only a very mild treatment of the bacterial pellet with formaldehyde, avoiding any modification that could occur during CPS extraction and purification. Compared to liquid phase NMR, HR-MAS NMR is simpler and faster, but the peak-to-peak resolution of CPS is lower.

When HR-MAS NMR was investigated for distinguishing serotypes 11A and 11E, the O- and N-acetyl methyl groups region (2.20 to 2.00 ppm), containing serotype specific NMR signals [8], did not prove a useful marker, since the serotype specific peaks were obscured by peaks associated with other cell surface components (i.e. proteins). However, the two serotypes could be readily distinguished with NMR signals in the anomeric proton region (5.80 to 4.40 ppm). Specifically, the H_4_^βGal-4,6OAc^ signal (at 5.6 ppm) proved to be a good marker for serotype 11A since it was missing in serotype 11E.

The results of HR-MAS NMR were in accordance with those of FCSA confirming that 23.5% of the previously identified serotype 11A isolates from invasive disease in our collection belonged to serotype 11E, while 11E was not identified among carriage isolates. This finding confirms results obtained in the only epidemiological study performed to date [15]. The detection of serotype 11E among invasive isolates only, suggests that suppression of β-Gal C_6_ O-acetylation promotes survival of pneumococcus during invasion and may represent a mechanism of immune evasion [15]. A recent study has demonstrated that ficolin-2, a host serum factor, recognizes specific O-acetylated epitopes of serotype 11A capsule polysaccharide thus inhibiting its invasiveness and likely selecting for serotype 11E micro-evolution during invasion [18].

All 11E isolates were characterized by the presence of *wcjE* mutations, including transposable element insertions, nucleotide duplications leading to a truncated protein, or missense mutations. With the exception of IS*1515* integrating in the same site in 3 isolates, the *wcjE* mutations were novel and unique indicating lack of clonal spread of 11E isolates.

Two isolates expressing serovariant 11Av were detected by FCSA only. One was an invasive isolate, while the other, originating from nasopharyngeal carriage, contained two different subpopulations, one expressing 11A and the other expressing 11Av, indicating that microevolution from 11A to 11Av can occur both in invasive disease and in carriage. HR-MAS NMR was less sensitive than FCSA in discriminating 11Av: in one case the isolate was identified as 11E, in the case of the mixed population the isolate was identified as 11A. Similar limitations were noted in the past for other serotyping methods, such as an ELISA-based serotyping method [13].

This study represents the first application of HR-MAS NMR to pneumococcal serotyping. HR-MAS NMR spectroscopy proved to be effective as a screening method to distinguish serotypes 11A and 11E and these results should encourage further investigations on the use of HR-MAS NMR in this field.

Since the pneumococcal capsule is the main target for protective antibodies and acetyl groups represent dominant epitopes [23], [24], the presence of these antigenic differences among circulating pneumococci must be taken into account in view of possible implications for the composition of new pneumococcal vaccines. In addition, minor serovariant populations can become common once the dominant capsule type is eradicated following active immunization. A well-known example is the emergence of serotype 6C following the decrease of serotypes 6A and 6B resulting from pneumococcal conjugate vaccine use [25]. In this scenario it is important to reappraise by innovative techniques the epidemiology of pneumococcal serotypes obtained by conventional serotyping.
